# Assessments and interventions on body functions, structures and activity to prepare adults with acute spinal cord injury or disease for participation: a scoping review

**DOI:** 10.3389/fresc.2024.1272682

**Published:** 2024-03-27

**Authors:** Sophie Irrgang, Sandra Himmelhaus, Kirstin Allek, Isabelle Debecker, Armin Gemperli, Karen Kynast, Anne von Reumont, Anke Scheel-Sailer

**Affiliations:** ^1^ Health Services and Clinical Care Group, Swiss Paraplegic Research, Nottwil, Switzerland; ^2^ Faculty of Health Sciences and Medicine, University of Lucerne, Lucerne, Switzerland; ^3^ Spinal Cord Injury Center, Zentralklinik Bad Berka, Bad Berka, Germany; ^4^ Occupational Therapy, REHAB Basel, Basel, Switzerland; ^5^ Spinal Cord Injury Center, Heidelberg University Hospital, Heidelberg, Germany; ^6^ Swiss Paraplegic Center, Nottwil, Switzerland

**Keywords:** spinal cord injury, acute phase, physiotherapy, occupational therapy, functioning

## Abstract

**Introduction:**

In the acute phase after a spinal cord injury or disease (SCI/D), various therapeutic assessments and interventions are applied with the goal of restoring structures, preventing complications and preparing the patient as best as possible for further activity and finally participation. The goal was to identify and evaluate the available evidence on assessments and interventions for body functions and structures to prepare adults with acute spinal cord SCI/D for activity and participation during the first 14 days.

**Methods:**

A scoping review was conducted. The search was performed on June 19, 2023 using the databases PubMed, PEDro, Cochrane library and Embase. These were screened for studies including patients with acute SCI/D and physiotherapeutic or occupational therapy assessments and interventions. Only studies in English or German published between 2012 and 2023 were included.

**Results:**

Twelve publications met the inclusion criteria, namely three systematic reviews, two randomized controlled trials, two observational studies and five clinical practice guidelines. Assessments as the Spinal Cord Independence Measure, as well as exercises such as daily passive mobilization of body structures against contractures were used in the entire population, while others were only applied in subgroups of SCI/D such as the Graded Redefined Assessment of Strength, Sensation and Prehension or functional electrical stimulation with and without additional movements. The methodological quality of the studies found varied greatly from good to very poor.

**Discussion:**

Heterogeneity in research design and study population as well as lack of high-quality studies do not cover the standard of clinical management in the acute phase and further comprehensive research is needed.

## 1 Introduction

A spinal cord injury or disease (SCI/D) can be a life-changing event for patients. Per definition, it can result in various disturbances in the sensory, motor or autonomic function and affects the patients physical, psychological and social well-being, ranging from nearly no impairment to the complete loss of functioning below the lesion level (1, 2). These different neurological impairments lead to complete different recovery patterns and thus to different care requirements depending on the patient (2). In addition, the different phases of care after SCI/D involve diverse healthcare professionals focusing on acute care and integrating rehabilitation as early as possible for restoring the patient's functional independence and autonomy and to avoid long-term consequences (3, 4). Considering the multitude of impairments and diverse needs, a key inquiry persists as to whether it is attainable to provide a more precise delineation of assessments and interventions during the acute phase after SCI/D.

On a structural and functional neurological level, the therapeutic goals in the acute phase are to minimize primary neurological damage and prevent secondary spinal cord injury due to hypoperfusion, ischemia and apoptotic or biochemical and inflammatory changes (5). To achieve these goals, an acute emergency medical, surgical, pharmacological or therapeutic management is performed through an interdisciplinary team (5). In addition to this acute treatment, early therapeutic rehabilitation may be possible during the acute phase to optimize long-term goals such as functional capacity and participation requiring an interprofessional team of physiotherapists, occupational therapists and sometimes speech therapists, social workers and psychologists (6, 7). These professions cover the individual need of the patient from a bio-psycho-social perspective based on the International Classification of Functioning, Disability and Health (ICF), a framework for describing and measuring health and disability at the individual and in a wider sense population levels (8, 9). The terms “activity” and “participation” are integral components of the ICF. “Activity” refers to specific tasks or actions an individual can perform, and “participation” reflects engagement in broader life situations (10, 11). These concepts are interconnected and contribute to a holistic understanding of an individual's health and well-being (8). Those components are universally used by healthcare workers at different settings including physicians, rehabilitation therapists, and social workers who examine the body structures, body functions and activity of patients with SCI/D. The specific goals of these therapies start within the acute phase after the onset of SCI/D and accompany patients during the whole course of the first rehabilitation after SCI/D (12). The interventions performed by these therapists usually cover restoration of structures, prevention of complications, access to assistive devices to maximize independence and best possible preparation for the patient's future activity and participation. Possible interventions include improving mobility or muscle strength, changing one's body position, independent locomotion or the use of assistive devices (13). The integration of assessments and interventions in the acute phase nevertheless often struggles due to patient's limited load capacity. Therefore, interventions cannot be performed in a standard way and the validation of assessments might be difficult due to reduced capacity of the patient. This leads to an additional uncertainty about the importance and recommendation of these baseline assessments as a starting point for the intervention.

Therefore, the type of assessments and therapies, the number and duration of treatments might play an important role during the acute phase in order to balance the effectiveness of therapies with the needs and capabilities of the patient and to adjust the type and intensity to the severity of the injury (6). However, an early safe use of a wide variety of therapies can lead to early improvement in the patient's activity for example through passive or strengthening exercises and prevention of complications as contractures might be an important goal (6).

Due to these different aspects and patients' situations, there still remain controversies about management strategies of SCI/D throughout de continuum of care starting in the acute phase. In this context, lack of standardization rehabilitation especially during the acute phase integrating the different needs of patients with acute SCI/D poses a major challenge (1). Standardization of therapeutic interventions during the acute phase with a focus on body functions and structures to prepare for activity participation based on scientific evidence might increase quality of care and consecutively the quality of life of patients living with a SCI/D.

In order to provide evidence for the conceptualization of rehabilitation strategies we aimed to determine the extent to which assessments and interventions during the first 14 days after SCI/D contribute to a successful course of rehabilitation, the following goal was set: To systematically search for therapeutic assessments and interventions focusing on body functions and structures to prepare for activity and participation during the acute phase in the first 14 days after newly acquired SCI/D and to evaluate the methodological quality of the available literature.

## 2 Methods

A scoping review was conducted, using the methodology outlined in the “Preferred Reporting Items for Systematic reviews and Meta-Analyses extension for Scoping Reviews” (PRISMA-ScR) guideline (14) (Appendix Table 1). This scoping review was not registered and therefore, no review protocol was created.

### 2.1 Literature search

A comprehensive literature research was conducted on December 16, 2022 followed by an update search on the June 19, 2023 using the databases PubMed, PEDro, Cochrane library and Embase. The basis for this search were *a priori* defined research question and underlying key questions (Table 1). The key questions relate to assessments and interventions on body functions and structures to prepare for activity and participation. Physio- and occupational therapeutic interventions in the acute phase, such as respiratory management or the supply of assistive devices, were excluded from the search. To find all relevant studies, both search terms and medical subject headings (MeSH) were used in the primary search. The following terms were used: “acute”, “therapeutic measures”, “therapeutic recommendation”, and the MeSH term for “spinal cord injury”, “acute disease”, “exercise therapy” and “treatment outcomes” (Appendix Table 2). The literature search was limited to year of publication from 2012 to 2023, English or German language and human studies. The search was completed by screening the references for additional literature and conducting a manual search.

**Table 1:** Key questions.

No.	Key question
1	Which assessments are used in the acute phase to measure the body functions and structures to prepare for activity and participation?
2	Which therapeutic interventions are used in the acute phase to maintain or improve muscle function?
3	Which interventions during the acute phase are suitable for the prevention of contractures and which are used to maintain joint mobility?
4	Which therapeutic interventions are used in the acute phase to improve hand function?
5	What supports the formation of the tenodesis grasp during the acute phase?

No., Number.

### 2.2 Eligibility criteria

The eligibility criteria of this scoping review were defined according to the PICOS framework and are presented in Table 2 (15). Hereby, systematic reviews, randomized controlled trials (RCTs), observational studies and clinical practice guidelines (CPG) were the desired study designs. The large number of different study designs was deliberately chosen to capture the full range of possible evidence. All studies that investigate or recommend assessments or interventions starting within the first 14 days after new SCI/D were included. Given that the acute phase is not uniformly defined in the SCI/D literature, we defined it as the first 14 days after the onset of SCI/D based on an expert consensus as part of the development of a S3 Guideline on diagnostics and therapy during the acute phase of SCI/D, which was officially registered on the website of the Association Working Group of Medical Societies (AWMF) (16). The consensus among the experts was based on the characterization of the early acute phase in the EMSCI study, which defined this phase as the first 15 days after an SCI/D (17). All patients with newly acquired SCI/D were included, regardless of their level and degree of paraplegia or etiology. Studies involving participants younger than 18 years, patients suffering from another acute or chronic musculoskeletal or neurological diseases, or animal studies were excluded. In case a study population include multiple diagnoses or stages of disease, 80% had to correspond to the inclusion criteria to be eligible.

**Table 2:** PICOS framework.

Criteria	Description
Population	Adults (≥18 years) with acute (until 14 days after the onset of the injury) spinal cord injury/disease
Interventions	Various interventions regarding physio- and occupational therapy in the acute phase
Comparators	No comparators
Outcomes	Therapeutic recommendations for use in acute care after newly acquired spinal cord injury
Study Design	Systematic reviews, randomized controlled trials, observational studies, clinical practice guidelines

### 2.3 Selection process

The selection of the retrieved studies was based on the predefined inclusion criteria (Appendix Table 3). To simplify the screening process, the tool *Rayyan* (18) was used. Two trained and blinded researchers (SI, SH) consecutively screened the studies for matching titles, abstracts and full texts. In case of ambiguity or disagreement during any stage of the screening process, an expert (ASS) was involved in the decision to include or exclude a study.

### 2.4 Analysis of study quality

To analyze the quality of the included literature, the checklists required by the AMWF were used (19). The quality of the available literature was evaluated with different tools. To assess the quality of systematic reviews and review articles, the MeaSurement Tool to Assess systematic Reviews 2 (AMSTAR 2) tool was used (20). RCTs were assayed by using Cochrane risk-of-bias tool for randomized controlled trials (RoB 2) (21). The quality of observational studies was assessed by using the Scottish Intercollegiate Guidelines Network (SIGN) checklists (22). Guidelines were reviewed using the Appraisal of Guidelines for Research & Evaluation II (AGREE II) tool. Scores over 70% was set to represent a good methodological quality in the according domain (23). These evaluations were conducted independently by two blinded reviewers (SI, SH) and were subsequently compared. Discrepancies between the two reviewers regarding the quality of the literature were discussed with a rehabilitation expert (ASS) experienced in the field of SCI/D.

### 2.5 Data collection and synthesis of results

The order of presentation of results is based on the revised evidence pyramid, so that systematic reviews come first, followed by RCTs, observational studies, and finally CPGs (24). To present the evidence on assessments and therapies in the acute phase of SCI/D, data was extracted from the included studies. The analysis of these results is based on the previously defined key questions.

The corresponding studies for each key question have been compiled in tabular form. This table displays information on author, year, design, population, number of studies or participants, assessments or interventions performed, outcome measurements, adverse events and subsequent findings and recommendations.

## 3 Results

### 3.1 Study selection

**Figure 1:**
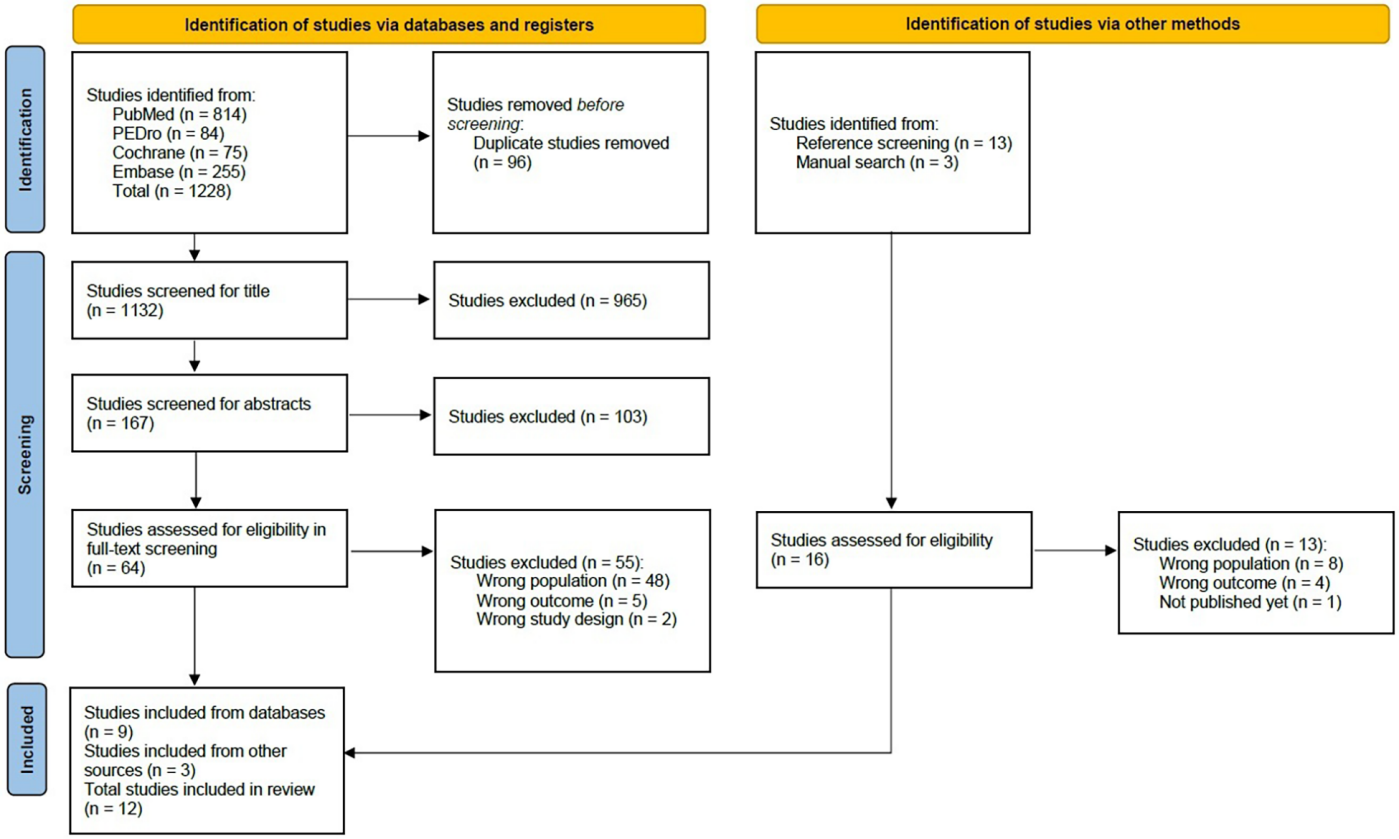
PRISMA flow diagram.

The comprehensive literature search resulted in 1,228 articles. After 96 duplicates had been removed, a total of 1,101 studies were screened for title and abstract. For the full-text screening, 64 studies remained. From these, nine studies were ultimately included in this scoping review. The primary factors leading to exclusion were either an extended period since SCI/D (<14 days) or incorrect study objectives, such as surgical or other invasive interventions. Additionally, a total of 16 further studies were found in the reference screening, three of which were additionally included. In total, twelve studies were included in this scoping review (25–36). The entire study selection process is outlined in Figure 1 (14).

### 3.2 Study characteristics

Three systematic reviews (25–27), two RCTs (28, 29), two observational studies (30, 31) and five CPGs (32–36) with recommendations for the PT and OT assessments and interventions were identified. The characteristics of the included studies are summarized in Table 3.

**Table 3:** Summary of the evidence.

First Author, Year	Design	Population	No. studies/participants	Intervention / Assessment	Extremities studied	Outcome measurements	Adverse events	Findings & Recommendations
Assessments								
Readdy (26)	Review Article	Acute cervical SCI	n.a.	n.a	n.a.	n.a.	n.a.	Clinical assessment: ASIA score, SCIM III
Bolliger (27)	Review Article	Complete / incomplete SCI	n.a	n.a	LE	n.a.	n.a.	Assessments for lower extremity function: ISNCSCI, WISCI II, SCIM III
Kalsi-Ryan (30)	Observational study	Traumatic cervical SCI	*n* = 60	GRASSP	UE	GRASSP, ISNCSCI (upper extremity motor score)	Not reported	GRASSP is a responsive and sensitive tool at all timepoints and can be applied during the first 14 days.
Oleson (31)	Observatio-nal study	Acute Tetraplegia	*n* = 65	CUE-Q	UE	ISNCSCI (upper extremity motor score), FIM, CUE-Q	Not reported	CUE-Q is a responsive and valid tool for assessing functional capacity of the arm and hand of persons with tetraplegia.
Walters (32)	Guideline	Acute SCI	n.a.	n.a	n.a.	n.a.	n.a.	Assessment of the functional status: SCIM (level 1)
Krylov (36)	Practice guideline	Acute SCI	n.a.	n.a	n.a.	n.a.	n.a.	Assessment of functional outcomes: FIM, Karnofsky scale
Muscle function								
Gomes-Osman (25)	Systematic Review	Acute traumatic SCI	*n* = 39	Electrical stimulation /neuroelectric devices, rehabilitation or combination of therapies including any of the above strategies	LE	FIM, WISCI II, walking distance, LEMS	Not reported	Greater improvements in outcomes of walking function when using robotic orthosis during locomotor training compared to overground walking.
Galea (29)	RCT	Acute complete/incomplete SCI (ASIA A-C, above Neurological level T12)	*n* = 24 (IG: 12, CG: 12)	IG: FES-cycling (Settings: 140 mA, 0.3–0.5 ms, 35 Hz, 30–40 rev cad) / CG: Passive cycling (passive cycling without resistance Or stimulation) /4x per week for 12 weeks, max. 60 min per session	LE	Muscle atrophy (MRI acquisition, image analysis), neurological function (ISNCSCI), anthropometric measurements (girth measure-ments with a cloth measuring tape), body composition (DEXA scans), quality of life, AE	Overall 10 SAEs (6 IG, 4 CG) and 128 AEs (159 IG, 122 CG). One SAE and one AE were related to the intervention.	Cycle ergometry is safe, and feasible early post-SCI. No significant benefit of one intervention over another (*p*-value > 0.05). Both interventions may be able to attenuate muscle atrophy compared with published data without intervention.
Fehlings (33)	Practice guideline	Acute SCI	n.a.	n.a.	n.a.	n.a.	n.a.	Therapy can be started as soon as the patients are medically stable.
Ginis (34)	Guideline	SCI	n.a.	n.a.	n.a.	n.a.	n.a.	Guidelines for patients with a chronic SCI may be appropriate for acute patients since there is a lack of evidence for the acute phase. Patients should consult HCP before starting with therapies.
Prevention of contractures & joint mobility								
Roquilly (35)	Guideline	Acute SCI	n.a.	n.a.	LE / UE	n.a.	n.a.	Application at least once a day of passive mobilisation of joints affected by motor deficit, positioning of joints in the opposite direction from the predictable deformation, application of an orthosis, manual muscle reinforcement. Stretching for at least 20 min per zone and completed by a posture orthosis and by bed or chair positioning.
Hand function								
Iwahashi (28)	RCT	Acute cervical SCI within 1 week after injury	*n* = 29 (IG: 15, CG: 14)	IG: orthotic TES administered using a neuroprothesis in addition to conventional training (5 min daily at 1 week, 15 min twice daily at 2 weeks, 20 min twice daily at 4 weeks) / CG: conventional training	UE	TPM of the fingers, Upper extremity motor score of the ISNCSCI, oedema measured at 1 week, 1 month and 3 months after injury in both hands	No adverse events	No significant between-group differences for TPM of the fingers, oedema and upper-extremity motor scores at 1 week, 1 month and 3 months after injury. TMP of the fingers tended to be lower in the control group. But TES can be started in the acute phase.
Fehlings (33)	Practice guideline	Acute SCI	n.a.	n.a.	UE	n.a.	n.a.	FES should be offered for patients with cervical SCI to improve hand and UE function.

SCI, spinal cord injury; n.a, not available; ASIA, American Spinal Injury Association; SCIM, spinal cord independence measure; LE, lower extremity; ISNCSCI, International standards for neurological classification of spinal cord injury; WISCI II, walking index for spinal cord injury II; GRASSP, Graded Redefined Assessment of Strength, Sensation and Prehension; UE, upper extremity; CUE-Q, Capabilities of Upper Extremity Questionnaire; FIM, functional independence measure; LEMS, lower extremity motor score; RCT, randomised controlled trial; IG, intervention group; CG, control group; FES, functional electrical stimulation; mA, milliampere; ms, millisecond; Hz, Hertz; rev cad, revolution cadence; MRI, magnetic resonance imaging; DEXA, dual energy x-ray absorptiometry; AE, adverse event; SAE, serious adverse event; HCP, health care professional; TES, therapeutic electric stimulation; TPM, total of passive movement.

### 3.3 Methodological quality assessment of the included studies

The systematic reviews, assessed with the AMSTAR 2 tool, achieved the following results out of 16 possible items: Gomes-Osman et al. (25) six, Readdy et al. (26) five and Bolliger et al. (27) two items (Appendix Table 4). Overall, all three systematic reviews were assessed with a critically low quality. The RCT of Iwahashi et al. (28) raised concerns regarding the risk of bias during its assessments using the RoB2 tool (Appendix Table 5). The RCT by Galea et al. (29) had a low risks of bias and was judged with a higher quality by the RoB2 tool (Appendix Table 6). The two observational studies (30, 31), assessed with the SIGN checklist, were classified having an overall acceptable methodological quality (Appendix Table 7). The CPG assessment relates to six domains of which Walters et al. (32) reached acceptable domain scores in three, Fehlings et al. (33) and Ginis et al. (34) each in five and Roquilly et al. (35) in two domains (Appendix Tables 8–11). As a final quality evaluation of the AGREE II tool these four CPGs were recommended for usage with modifications. Krylov et al. (36) were not able to reach a domain score from over 70% and was therefore not recommended for further usage (Appendix Table 12).

### 3.4 Synthesis of the results

The synthesis of the results was based on the key questions presented in Table 1.

The identified interventions encompass various outcomes, objectives and therapeutic approaches. However, this variability illustrates that even in the acute phase of SCI/D, a spectrum of interventions may be considered to improve body functions and structures to prepare for activity and participation throughout the continuum of care.

The study characteristics and a summary of the evidence is displayed in Table 3. Four studies originated from the United States of America (25, 26, 31, 32). Three studies were done in Canada (30, 33, 34) and one each in Russia (36), Switzerland (27), France (35), Australia (29) and Japan (28) (Table 3).

#### 3.4.1 Which assessments are used in the acute phase to measure the body functions and structures to prepare for activity and participation?

Bolliger et al. (27) elaborated in their review that there are different lower extremity assessments throughout different disease phases and patient types. For all types of SCI/D, they suggest using the International Standards for Neurological Classification of Spinal Cord Injuries (ISNCSCI), the Walking Index for Spinal Cord Injuries II (WISCI II) and the Spinal Cord Independence Measure III (SCIM III) (27). The SCIM or the third version of the SCIM (SCIM III) are also proposed by Readdy et al. (26) and Walters et al. (32) for use in the acute phase of SCI/D. Walters et al. (32) rate the level of this recommendation as level 1, reflecting a strong recommendation.

To classify the general status of the patient and their injury or illness, Readdy et al. (26) suggest the American Spinal Injury Association (ASIA) score, which is meanwhile known as ISNCSCI.

The observational studies from Kalsi-Ryan et al. (30) and Oleson et Marino (31) both looked at the responsiveness of assessments of hand and arm function. Kalsi-Ryan et al. (30) proposed the use of the Graded Redefined Assessment of Strength, Sensation and Prehension (GRASSP) from the tenth day after the onset of a traumatic cervical SCI onwards. They found this tool to be responsive and sensitive in their study with 60 participants (30). Oleson et Marino (31) documented a high responsiveness and validity of the capabilities of upper extremity questionnaire (CUE-Q), which is why they suggest the use to assess the upper extremity function in patients with an acute traumatic tetraplegia.

Krylov et al. (36) were recommending in their CPG to use the Functional Independence Measure (FIM) to assess functional outcomes instead of the SCIM. They also consider the use of the Karnofsky scale for functional assessments during the first 14 days after the onset of a SCI/D (36).

#### 3.4.2 Which therapeutic interventions are used in the acute phase to maintain or improve muscle function?

The systematic review of Gomes-Osman et al. (25) found positive effects of robotic training on muscle function in the lower extremities and ADLs and no adverse events resulting from the interventions respecting the patient's performance and neurological conditions in patients with a traumatic SCI. They also observed a positive effect of locomotion training with a robotic orthosis on muscle and walking function (25).

The RCT of Galea et al. (29) compared functional electrical stimulation (FES)-cycling with passive cycling, both conducted on a leg cycle ergometer, over a period of 12 weeks, starting during the first 14 days after the onset of a SCI/D. Included were 24 patients with a motor complete or incomplete SCI ASIA A-C above the neurological level of T12. Looking at the primary outcome of muscle atrophy, the authors did not find a significant benefit of one intervention over another. Nevertheless, they stated, that leg cycle ergometry is a safe and feasible therapy early after the onset of a SCI/D. Furthermore, both interventions tested may be able to attenuate muscle atrophy and therefore improve muscle function when comparing the study data with other published data without any intervention (29).

In their CPG, Fehlings et al. (33) recommended starting PT with patients when they are medically stable and can tolerate the required intensity of therapy. However, the exact intensity or the extremities used in the therapy were not defined more precisely. The expected outcomes of the recommendation are improved neurological status, ADLs and quality of live. The strength of this recommendation was rated as weak by the guideline authors (33).

Ginis et al. (34) noted in their CPG that other exercise CPGs for patients with chronic SCI/D may be appropriate for use in acute patients. The authors' recommendation lacked specification regarding the specific body regions to be targeted for training. The uncertainty in this recommendation stems from the fact that to date, there is too little scientific data and evidence concerning exercises during the acute phase of SCI/D. The authors stated that the patient should consult a healthcare professional before performing exercises to improve muscle function (34).

#### 3.4.3 Which interventions during the acute phase are suitable for the prevention of contractures and which are used to maintain joint mobility?

As an expert opinion in their CPG, Roquilly et al. (35) suggested the following interventions to reduce limb spasticity and contractures: (I) rehabilitation and passive mobilization of body structures affected by the motor deficit, (II) positioning of joints in the opposite direction from the predictable deformation, (III) application of an orthosis, (IV) manual muscle reinforcement. These interventions should be performed at least once a day right from the beginning on. Furthermore, they suggested that stretching should be performed for at least 20 min per zone and be completed by a posture orthosis and by bed or chair positioning to prevent and correct predictable deformities (35).

#### 3.4.4 Which therapeutic interventions are used in the acute phase to improve hand function?

The RCT of Iwahashi et al. (28) included 29 patients with an acute cervical SCI. They compared a combination of orthotic therapeutic electric stimulation (TES) with conventional training and simple conventional training, starting within the first week after the onset of a SCI/D. Total of passive movement (TPM) of the fingers, upper-extremity motor score and hand edema at 1 week, 1 month and 3 months after the onset were measured. No significant between-group differences were found in all outcome measurements. There was a slight negative trend in the TPM of the fingers in the control group, which tended to be lower. Nevertheless, the authors stated that TES is a possible therapy that can be started in the acute phase, as there were no adverse events during their study (28).

The CPG by Fehlings et al. (33) indicated that FES should be offered as part of therapy. The strength of this recommendation was rated as weak, as little evidence supports this recommendation and no therapy parameters or intensities were given (33).

#### 3.4.5 What supports the formation of the tenodesis grasp during the acute phase?

No studies about possible therapies to support the formation of a tenodesis grasp during the acute phase were found in the literature search.

## 4 Discussion

### 4.1 Summary of the evidence

Three studies used the SCIM III as a standard assessment for all patients with SCI/D already during the acute phase (26, 27, 32), while the FIM is only given as another functional assessment by Krylov et al. (36). In addition, other assessments such as the GRASSP or the WISCI II were considered reliable assessments early after SCI/D (27, 30). We found four publications addressing the question of improving muscle function in patients within the first 14 days after the onset of a SCI/D. The results of these studies included unspecified PT or OT once the patient is medically stable and robotic assisted training or FES-cycling to improve activity and mobility (25, 29, 33, 34). To prevent contractures and maintain joint mobility, one CPG suggested the application of different interventions like passive mobilization of body structures at least once a day during the acute phase (35). Regarding the question of the improvement of hand function, two studies recommended FES and orthotic TES (28, 33).

During the acute rehabilitation, the SCIM III was the most used assessment tool in the interprofessional team (37). The SCIM III can be used over a long period of time during therapy. For instance, Hodel et al. (38) found in their longitudinal analysis that the SCIM III can be used for the first time eleven days after admission to rehabilitation and up to 132 days afterwards. In addition, the SCIM III was already used for some patients during their time in the intensive care unit (38). Ideally, the time in the intensive care unit (ICU) is already spent in a SCI/D-specific center, to offer the specific therapies through an experienced interprofessional that is also trained in performing these specific assessments. Though, the average time of admission from the acute hospital to a SCI/D-specific center is 16.2 days (39). The ISNCSCI, proposed by Bolliger et al. (27), should be conducted within the first 40 days after the onset of SCI/D, according to its 2019 revision (40). According to a Swiss study, 59% of the included participants were assessed with the ISNCSCI within the suggested time frame of 40 days (41). Furthermore, the assessment of the ISNCSCI may take up to 60 min (42), which, depending on the patient, can pose challenges in the execution during the acute phase. Completing the ISNCSCI is not only time-consuming, but also requires specialized training. According to the study by Schuld et al. (43), practitioners, such as physicians or therapists, benefit from several trainings to complete the assessment correctly. This highlights that although this assessment is regularly used in a SCI/D specific rehabilitation, the implementation still has room for improvement. Although both assessments are commonly used in the acute phase, it is important to distinguish their purpose carefully. The ISNCSCI describes the sensory and motor impairment, as well as the neurological level and completeness of the injury. However, it does not include functional testing. The SCIM III, on the other hand, tests different functional areas such as self-care or patient's mobility abilities. Since both the SCIM and the ISNCSCI are used in the acute phase as well as in the rehabilitative phase, they can be employed to evaluate the patient's development over time. When used right from the beginning, it becomes possible to track the improvements, particularly in the functional areas, throughout the continuum of care as there is a baseline measurement.

It was found that there is an indication of a relationship between PT interventions and improved participation in daily life in the long term and interventions help to improve impairments such as weakness and limited joint mobility (44, 45). However, there is still uncertainty about the effectiveness of the different interventions and intensities focusing on strengthening or prevention of complications in the long term (44). When examining interventions for improving hand function in the acute phase, there is evidence supporting treatment with FES for functional independence of the upper extremity and functional exercises in rehabilitation. But also in this intervention, not only a specialized training but also a cost-intensive technical equipment is needed. To support the formation of a tenodesis grasp in the post-acute phase, a combination of splinting and passive movements is suggested during the course of rehabilitation (46), but uncertainty remains if the tenodesis grasp is desirable at all and at what time the formation should be commenced. When trying to transfer therapies from later rehabilitation stages to the acute phase setting, feasibility of therapies is a major issue. Some add-on therapies like robot-assisted treadmill training will not be feasible for most patients in the first 14 days of a SCI/D while some subgroups of patient can integrate this treatment and achieve their goals on walking and mobility even faster. Nevertheless, the lack of evidence in the acute phase and the complex requirements remain a challenge for clinical practice.

Regarding the results of this review, a discrepancy between established therapies in the clinical management during the acute phase of SCI/D and evidence-based therapies has emerged. Many used interventions are derived from CPG recommendations based on expert opinion, which often lack methodological quality. In various therapeutic textbooks, different therapeutic interventions are describe based on expert knowledge and established treatment concepts in specialized SCI centers without any scientific evidence. Consequently, there is currently a lack of more precise information and treatment parameters for possible interventions, highlighting the importance of better-quality evidence with clearer treatment parameters. Overcoming this challenges will ultimately aid in the development of CPG recommendations and thus provide a more accurate representation of the therapeutic interventions used in real world clinical practice (47).

### 4.2 Limitations of the study

Since the search was limited to German- and English-language studies, it is possible that studies in other languages could not be included in this scoping review. Furthermore, the search period was limited to the years 2012 to 2023 to ensure that the results are up to date. However, it is possible that relevant studies published before 2012 were not included in this review.

The methodological quality of the studies varied greatly from good quality (34) to very poor quality (27, 36). The poor quality of some studies raises questions about the significance of the results found, which can have an influence on their interpretation. Therefore, it is difficult to consider the recommendation by Krylov et al. (36) to use the FIM as an assessment for patients with SCI/D as a reliable finding of this review, as the quality of this CPG was rated as very poor.

Apart from the insufficient quality of the studies, the varying definitions of the acute phase after a SCI/D posed challenges in the evaluation of the studies. For instance, in the study by Gomes-Osman et al. (25), the acute phase is defined as minutes to six months after the onset of a SCI/D. In other studies, no clear definition of the acute phase after SCI/D was given (33, 36). These different definitions made a precise search challenging and a clear interpretation of the study results difficult. Since the included studies describe different clinical pictures and levels of paralysis, there is limited generalizability of the presented results. Depending on the clinical picture and its severity, the therapies applied differ greatly, which is why the results of the individual studies cannot be transferred to the entire population of patients with SCI/D.

### 4.3 Implementation and further research

The results of this review showed that early assessments and therapeutic interventions during the acute phase after SCI/D are safe and can be integrated in the clinical management of any trauma center in the acute phase to optimize body functions and structures to prepare for activity and participation for a long-term perspective. Existing evidence rather focuses on new interventions which often are technically supported and may often be considered as add-on therapies. Yet, established clinical practices or routines are not captured in a scientifically based manner. Due to the lack of high-quality evidence, the recommendations of the CPGs are mostly based on expert opinion. International multicenter observational studies should focus on therapy intensity or training parameters, age groups of the patients or the disease-related subgroups and outcome measurements in all stages to understand the benefit of therapeutic interventions in the first 14 days of SCI/D. The scientific community should agree on common definitions of disease stages and accordingly indicate the time since injury in studies. It might be important to include the aspects of safety and the competencies of therapeutic health care professionals to develop a strong recommendation for basic therapy interventions adapted to the functioning status of the individual patient.

## 5 Conclusion

The SCIM III, the ISNCSCI and the GRASSP version 2 can be used as assessments in the early phase, serving as starting points for outcomes measurements in the continuum of rehabilitation and should be conducted as soon as the individual patients is able to perform the assessment. Therapies in the first 14 days after SCI/D are important to avoid complications, to support functioning and to guarantee a basis for the subsequent participation of patients with newly acquired SCI/D. However, due to the low quality and heterogeneity of studies on assessments and therapies in the acute phase, no specific and patient-tailored recommendation can be made. Observational studies might be the next step to further substantiate therapeutic assessments and interventions during the acute phase after SCI/D.

## Data Availability

The original contributions presented in the study are included in the article/Supplementary Material, further inquiries can be directed to the corresponding author.
