# Supplementary material for: Assessments and interventions on body functions, structures and activity to prepare adults with acute spinal cord injury or disease for participation: a scoping review
Source: Front Rehabil Sci. 2024 Mar 27;5:1272682. doi: 10.3389/fresc.2024.1272682 (PMC11004486; doi:10.3389/fresc.2024.1272682)
Supplement: Supplementary file 1 [file Table1.docx]

Supplementary Material

Assessments and Interventions on Body Functions, Structures and Activity to Prepare Adults with Acute Spinal Cord Injury or Disease for Participation : A Scoping Review

Sophie Irrgang*, Sandra Himmelhaus, Kirstin Allek, Isabelle Debecker, Armin Gemperli, Karen Kynast, Anne von Reumont, Anke Scheel-Sailer

*** Correspondence:** Sophie Irrgang: sophie.irrgang@paraplegie.ch

# Tables

Appendix Table 1. PRISMA extension for Scoping Reviews (PRISMA-ScR) checklist

| **SECTION** | **ITEM** | **PRISMA-ScR CHECKLIST ITEM** | **REPORTED ON PAGE #** |
| --- | --- | --- | --- |
| **TITLE** | | | |
| Title | 1 | Identify the report as a scoping review. | Page 1 |
| **ABSTRACT** | | | |
| Structured summary | 2 | Provide a structured summary that includes (as applicable): background, objectives, eligibility criteria, sources of evidence, charting methods, results, and conclusions that relate to the review questions and objectives. | Page 1 |
| **INTRODUCTION** | | | |
| Rationale | 3 | Describe the rationale for the review in the context of what is already known. Explain why the review questions/objectives lend themselves to a scoping review approach. | Page 2 |
| Objectives | 4 | Provide an explicit statement of the questions and objectives being addressed with reference to their key elements (e.g., population or participants, concepts, and context) or other relevant key elements used to conceptualize the review questions and/or objectives. | Page 2 |
| **METHODS** | | | |
| Protocol and registration | 5 | Indicate whether a review protocol exists; state if and where it can be accessed (e.g., a Web address); and if available, provide registration information, including the registration number. | Page 2 |
| Eligibility criteria | 6 | Specify characteristics of the sources of evidence used as eligibility criteria (e.g., years considered, language, and publication status), and provide a rationale. | Page 3 |
| Information sources* | 7 | Describe all information sources in the search (e.g., databases with dates of coverage and contact with authors to identify additional sources), as well as the date the most recent search was executed. | Page 2 |
| Search | 8 | Present the full electronic search strategy for at least 1 database, including any limits used, such that it could be repeated. | Page 3 & Appendix Table 2 |
| Selection of sources of evidence† | 9 | State the process for selecting sources of evidence (i.e., screening and eligibility) included in the scoping review. | Page 3 |
| Data charting process‡ | 10 | Describe the methods of charting data from the included sources of evidence (e.g., calibrated forms or forms that have been tested by the team before their use, and whether data charting was done independently or in duplicate) and any processes for obtaining and confirming data from investigators. | Page 3 |
| Data items | 11 | List and define all variables for which data were sought and any assumptions and simplifications made. | Page 3 |
| Critical appraisal of individual sources of evidence§ | 12 | If done, provide a rationale for conducting a critical appraisal of included sources of evidence; describe the methods used and how this information was used in any data synthesis (if appropriate). | Page 3 |
| Synthesis of results | 13 | Describe the methods of handling and summarizing the data that were charted. | Page 3 |
| **RESULTS** | | | |
| Selection of sources of evidence | 14 | Give numbers of sources of evidence screened, assessed for eligibility, and included in the review, with reasons for exclusions at each stage, ideally using a flow diagram. | Page 3-4 |
| Characteristics of sources of evidence | 15 | For each source of evidence, present characteristics for which data were charted and provide the citations. | Page 4-8 |
| Critical appraisal within sources of evidence | 16 | If done, present data on critical appraisal of included sources of evidence (see item 12). | Page 4 & Appendix Table 3-12 |
| Results of individual sources of evidence | 17 | For each included source of evidence, present the relevant data that were charted that relate to the review questions and objectives. | Page 4-8 & Table 3 |
| Synthesis of results | 18 | Summarize and/or present the charting results as they relate to the review questions and objectives. | Page 4-8 & Table 3 |
| **DISCUSSION** | | | |
| Summary of evidence | 19 | Summarize the main results (including an overview of concepts, themes, and types of evidence available), link to the review questions and objectives, and consider the relevance to key groups. | Page 8-9 |
| Limitations | 20 | Discuss the limitations of the scoping review process. | Page 9 |
| Conclusions | 21 | Provide a general interpretation of the results with respect to the review questions and objectives, as well as potential implications and/or next steps. | Page 9 |
| **FUNDING** | | | |
| Funding | 22 | Describe sources of funding for the included sources of evidence, as well as sources of funding for the scoping review. Describe the role of the funders of the scoping review. | Page 10 |

Appendix Table 2. Search Strategy

| **Pubmed** |
| --- |
| (("Spinal Cord Injuries"[Mesh]) OR (Paraplegia[Mesh]) OR (Quadriplegia[Mesh])) AND (("Acute Disease"[Mesh]) OR (acut*) OR (earl*) OR (urge*) OR (emerg*)) AND ((therapeutic intervention*) OR (therapeutic measures) OR (therap*) OR (Physical Therapy Modalities[Mesh]) OR (Exercise Therapy[Mesh]) OR (Occupational Therapy[Mesh]) OR (Treatment Outcome[Mesh])) AND ((therapeutic recommendation*) OR (Muscle Function[Mesh]) OR (prevention of contractures) OR (Range of Motion, Articular[Mesh]) OR (musculoskeletal limitation*) OR (functional hand) OR (hand function) OR (tenodesis grasp) OR (tenodesis hand) OR (Treatment Outcome[Mesh]) OR (Outcome Assessment, Health Care*[Mesh])) AND ((humans[Filter]) AND (English[Filter] OR german[Filter]) AND (2012:2023[pdat])) |
| **PEDro** |
| Spinal cord injury AND therapy (since 2012) |
| **Cochrane library** |
| #1 MeSH descriptor: [Spinal Cord Injuries] explode all trees  #2 MeSH descriptor: [Paraplegia] explode all trees  #3 MeSH descriptor: [Quadriplegia] explode all trees  #4 MeSH descriptor: [Acute Disease] explode all trees  #5 ((acut*) OR (earl*) OR (urge*) OR (emerg*)):ti,ab,kw  #6 ((therapeutic intervention*) OR (therapeutic measures) OR (therap*)):ti,ab,kw  #7 MeSH descriptor: [Physical Therapy Modalities] explode all trees  #8 MeSH descriptor: [Exercise Therapy] explode all trees  #9 MeSH descriptor: [Occupational Therapy] explode all trees  #10 MeSH descriptor: [Treatment Outcome] explode all trees  #11 ((therapeutic recommendation*) OR (prevention of contractures) OR (musculoskeletal limitation*) OR (functional hand) OR (hand function) OR (tenodesis grasp) OR (tenodesis hand)):ti,ab,kw  #12 MeSH descriptor: [Range of Motion, Articular] explode all trees  #13 MeSH descriptor: [Outcome Assessment, Health Care] explode all trees 1  #14 (#1 OR #2 OR #3) AND (#4 OR #5) AND (#6 OR #7 OR #8 OR #9 OR 310) AND (#11 OR #12 OR #13) with Publication Year from 2012 to 2023, with Cochrane Library publication date Between Jan 2012 and Jun 2023, in Trials |
| **Embase** |
| ('spinal cord injuries'/exp OR 'spinal cord injuries' OR 'paraplegia'/exp OR 'paraplegia' OR 'quadriplegia'/exp OR 'quadriplegia') AND ('acute disease'/exp OR 'acute disease' OR acut* OR earl* OR urge* OR emerg*) AND ('therapeutic intervention*' OR 'therapeutic measures' OR therap* OR 'physical therapy modalities'/exp OR 'physical therapy modalities' OR 'exercise therapy'/exp OR 'exercise therapy' OR 'occupational therapy'/exp OR 'occupational therapy') AND ('therapeutic recommendation*' OR 'muscle function'/exp OR 'muscle function' OR 'prevention of contractures' OR 'range of motion, articular'/exp OR 'range of motion, articular' OR 'musculoskeletal limitation*' OR 'functional hand' OR 'hand function' OR 'tenodesis grasp' OR 'tenodesis hand' OR 'treatment outcome'/exp OR 'treatment outcome' OR 'outcome assessment, health care*') AND ([english]/lim OR [german]/lim) AND [humans]/lim AND [embase]/lim AND ([cochrane review]/lim OR [systematic review]/lim OR [randomized controlled trial]/lim OR 'controlled clinical trial'/exp OR 'controlled clinical trial') AND [2012-2023]/py |

Appendix Table 3. Eligibility Criteria

| Inclusion Criteria | Exclusion Criteria |
| --- | --- |
| Patients with spinal cord injury or disease | Additional chronic musculoskeletal disease or other neurological diseases |
| Acute care until 14 days after the onset | Rehabilitation after 14 days |
| Various interventions and assessments regarding physio-  and occupational therapy | Other |
| Randomised controlled trials, observational studies, systematic reviews, review articles, guidelines | All other types of studies |
| Human studies | Animal studies |
| In English or German | All other languages |
| Studies published between 2012-2023 | Studies published before 2012 |

Appendix Table 4. Quality assessment of included systematic reviews according to AMSTAR II

| Reference | Item 1 | Item 2 | Item 3 | Item 4 | Item 5 | Item 6 | Item 7 | Item 8 | Item 9 | Item 10 | Item 11 | Item 12 | Item 13 | Item 14 | Item 15 | Item 16 | Overall quality |
| --- | --- | --- | --- | --- | --- | --- | --- | --- | --- | --- | --- | --- | --- | --- | --- | --- | --- |
| Bolliger et al. | 🗸 | 🗴 | 🗴 | 🗴 | 🗴 | 🗴 | 🗴 | 🗴 | 🗴 | 🗴 | 🗴 | 🗴 | 🗴 | 🗴 | 🗴 | 🗸 | Critically low |
| Readdy et al. | 🗸 | 🗴 | 🗴 | 🗴 | 🗸 | 🗸 | 🗴 | 🗸 | 🗴 | 🗴 | 🗴 | 🗴 | 🗴 | 🗴 | 🗴 | 🗸 | Critically low |
| Gomes-Osman et al. | 🗸 | ½ | 🗸 | 🗴 | 🗸 | 🗴 | 🗴 | ½ | 🗸 | 🗴 | 🗴 | 🗴 | 🗴 | 🗸 | 🗴 | 🗸 | Critically low |
| 🗸: all items were adequately addressed; ½: items were partially addressed; 🗴: one or more items were not adequately addressed | | | | | | | | | | | | | | | | | |

Appendix Table 5. Quality assessment of Iwahashi et al. according to RoB 2

| **Iwahashi et al.** |  |  |  |  |  |
| --- | --- | --- | --- | --- | --- |
| Domain | Item 1 | Item 2 | Item 3 | Item 4 | Risk of bias |
| 1 – Randomization process | No | Yes | No | - | Some concerns |
| 2 – Deviations from the intended intervention | NI | Probably  yes | No | - | Low |
| 3 – Missing outcome data | Yes | - | - | - | Low |
| 4 – Measurement of the outcome | No | No | Yes | Probably  no | Low |
| 5 – Selection of the reported results | NI | Probably  no | No | - | Some concerns |
| Overall Risk of bias |  |  |  |  | Some concerns |
| NI: no information; -: not applicable | | | | |  |

Appendix Table 6. Quality assessment of Galea et al. according to RoB 2

| **Galea et al.** |  |  |  |  |  |
| --- | --- | --- | --- | --- | --- |
| Domain | Item 1 | Item 2 | Item 3 | Item 4 | Risk of bias |
| 1 – Randomization process | Yes | Yes | No | - | Low |
| 2 – Deviations from the intended intervention | Yes | Yes | Probably  no | - | Low |
| 3 – Missing outcome data | Yes | - | - | - | Low |
| 4 – Measurement of the outcome | No | No | No | - | Low |
| 5 – Selection of the reported results | Yes | No | No | - | Low |
| Overall Risk of bias |  |  |  |  | Low |
| -: not applicable |  |  |  |  |  |

Appendix Table 7. Quality assessment of included observational studies according to SIGN

| Reference | Item 1 | Item 2 | Item 3 | Item 4 | Item 5 | Item 6 | Item 7 | Item 8 | Item 9 | Item 10 | Item 11 | Item 12 | Item 13 | Item 14 | Item 15 | Item 16 | Item 17 |
| --- | --- | --- | --- | --- | --- | --- | --- | --- | --- | --- | --- | --- | --- | --- | --- | --- | --- |
| Kalsi-Ryan et al. | 🗸 | 🗸 | d.n.a. | d.n.a. | 10.17% | d.n.a. | 🗸 | d.n.a. | d.n.a. | 🗸 | d.n.a. | 🗸 | 🗴 | 🗴 | + | 🗸 | 🗸 |
| Oleson et Marino | 🗸 | d.n.a. | d.n.a. | d.n.a. | 35.38% | d.n.a. | 🗸 | d.n.a. | d.n.a. | 🗸 | d.n.a. | 🗸 | 🗴 | 🗴 | + | 🗸 | 🗸 |
| 🗸: item was adequately adressed; 🗴: item was not adequately addressed; d.n.a.: does not apply; +: overall acceptable methodological quality | | | | | | | | | | | | | | | | | |

Appendix Table 8. Quality assessment of Walters et al. according to AGREE II

| **Walters et al.** | | | | |
| --- | --- | --- | --- | --- |
| AGREE II domain | Points | Maximum points | Minimum points | Domain score |
| 1. Scope and purpose | 14 | 21 | 3 | 61% |
| 2. Stakeholder involvement | 10 | 21 | 3 | 39% |
| 3. Rigour of development | 22 | 56 | 8 | 29% |
| 4. Clarity of presentation | 16 | 21 | 3 | 72% |
| 5. Applicability | 23 | 28 | 4 | 79% |
| 6. Editorial independence | 14 | 14 | 2 | 100% |
| Overall | 4 | 7 | 1 | - |
| *Recommendation for use: Yes, with modifications* | | | | |

Appendix Table 9. Quality assessment of Fehlings et al. according to AGREE II

| **Fehlings et al.** | | | | |
| --- | --- | --- | --- | --- |
| AGREE II domain | Points | Maximum points | Minimum  points | Domain score |
| 1. Scope and purpose | 21 | 21 | 3 | 100% |
| 2. Stakeholder involvement | 16 | 21 | 3 | 72% |
| 3. Rigour of development | 52 | 56 | 8 | 92% |
| 4. Clarity of presentation | 18 | 21 | 3 | 83% |
| 5. Applicability | 19 | 28 | 4 | 63% |
| 6. Editorial independence | 13 | 14 | 2 | 92% |
| Overall | 6 | 7 | 1 | - |
| *Recommendation for use: Yes, with modifications* | | | | |

Appendix Table 10. Quality assessment of Ginis et al. according to AGREE II

| **Ginis et al.** | | | | |
| --- | --- | --- | --- | --- |
| AGREE II domain | Points | Maximum  points | Minimum points | Domain score |
| 1. Scope and purpose | 20 | 21 | 3 | 94% |
| 2. Stakeholder involvement | 19 | 21 | 3 | 89% |
| 3. Rigour of development | 47 | 56 | 8 | 81% |
| 4. Clarity of presentation | 14 | 21 | 3 | 61% |
| 5. Applicability | 22 | 28 | 4 | 75% |
| 6. Editorial independence | 12 | 14 | 2 | 83% |
| Overall | 6 | 7 | 1 | - |
| *Recommendation for use: Yes, with modifications* | | | | |

Appendix Table 11. Quality assessment of Roquilly et al. according to AGREE II

| Roquilly et al. | | | | |
| --- | --- | --- | --- | --- |
| AGREE II domain | Points | Maximum points | Minimum points | Domain score |
| 1. Scope and purpose | 16 | 21 | 3 | 72% |
| 2. Stakeholder involvement | 10 | 21 | 3 | 39% |
| 3. Rigour of development | 29 | 56 | 8 | 44% |
| 4. Clarity of presentation | 19 | 21 | 3 | 89% |
| 5. Applicability | 6 | 28 | 4 | 8% |
| 6. Editorial independence | 9 | 14 | 2 | 58% |
| Overall | 5 | 7 | 1 | - |
| *Recommendation for use: Yes, with modifications* | | | | |

Appendix Table 12. Quality assessment of Krylov et al. according to AGREE II

| **Krylov et al.** | | | | |
| --- | --- | --- | --- | --- |
| AGREE II domain | Points | Maximum  points | Minimum points | Domain score |
| 1. Scope and purpose | 4 | 21 | 3 | 6% |
| 2. Stakeholder involvement | 6 | 21 | 3 | 17% |
| 3. Rigour of development | 15 | 56 | 8 | 15% |
| 4. Clarity of presentation | 15 | 21 | 3 | 67% |
| 5. Applicability | 6 | 28 | 4 | 8% |
| 6. Editorial independence | 2 | 14 | 2 | 0% |
| Overall | 2 | 7 | 1 | - |
| *Recommendation for use: No* | | | | |
